# Hepatoprotective activity of *Sonchus asper* against carbon tetrachloride-induced injuries in male rats: a randomized controlled trial

**DOI:** 10.1186/1472-6882-12-90

**Published:** 2012-07-09

**Authors:** Rahmat A Khan, Muhammad R Khan, Sumaira Sahreen, Naseer Ali Shah

**Affiliations:** 1 Department of Biotechnology, Faculty of Biological Sciences, University of Science and Technology Bannu, Khyber Pakhtunkhwa, Pakistan; 2 Department of Biochemistry, Faculty of Biological Sciences, Quaid-I-Azam University Islamabad, Islamabad, Pakistan; 3 Botanical Sciences Division, Pakistan Museum of Natural History, Islamabad, Pakistan

**Keywords:** Carbon tetrachloride, *Sonchus asper*, Liver histopathology, Antioxidant, Lipid peroxidation, Liver enzymes

## Abstract

**Abstract:**

**Background:**

*Sonchus asper* (SAME) is used as a folk medicine in hepatic disorders. In this study, the hepatoprotective effects of the methanol extract of SAME was evaluated against carbon tetrachloride (CCl_4_)-induced liver injuries in rats.

**Methods:**

To evaluate the hepatoprotective effects of SAME, 36 male Sprague–Dawley rats were equally divided into 6 groups. Rats of Group I (control) were given free access to approved feed and water. Rats of Group II were injected intraperitoneally with CCl_4_ (3 ml/kg) as a 30% solution in olive oil (v/v) twice a week for 4 weeks. Animals of Groups III (100 mg/kg) and IV (200 mg/kg) received SAME, whereas those of Group V were given silymarin *via* gavage (100 mg/kg) after 48 h of CCl_4_ treatment. Group VI received SAME (200 mg/kg) twice a week for 4 weeks without CCl_4_ treatment. Various parameters, such as the serum enzyme levels, serum biochemical marker levels, antioxidant enzyme activities, and liver histopathology were used to estimate the hepatoprotective efficacy of SAME.

**Results:**

The administration of SAME and silymarin significantly lowered the CCl_4_-induced serum levels of hepatic marker enzymes (aspartate aminotransferase, alanine aminotransferase, and lactate dehydrogenase), cholesterol, low-density lipoprotein, and triglycerides while elevating high-density lipoprotein levels. The hepatic contents of glutathione and activities of catalase, superoxide dismutase, glutathione peroxidase, glutathione S-transferase, and glutathione reductase were reduced. The levels of thiobarbituric acid-reactive substances that were increased by CCl_4_ were brought back to control levels by the administration of SAME and silymarin. Liver histopathology showed that SAME reduced the incidence of hepatic lesions induced by CCl_4_ in rats.

**Conclusion:**

SAME may protect the liver against CCl_4_-induced oxidative damage in rats.

## Background

Hepatotoxicity is the most widespread pathology worldwide, representing up to 83% of all cases. Hepatitis, viral infections, food additives, alcohol, toxic industrial chemicals, and air and water pollutants are the major risk factors of liver toxicity. There is increasing evidence that free radicals and reactive oxygen species play a crucial role in various steps that initiate and regulate the progression of liver diseases independently of the original agent [1]. Carbon tetrachloride (CCl_4_) is a potent environmental hepatotoxin [2] that, in addition to hepatic problems, causes dysfunction of the kidneys, lungs, testis, brain, and blood by generating free radicals [3,4].

CCl_4_ requires bioactivation in phase I of the cytochrome P450 system to form the reactive metabolic trichloromethyl radical (·CCl_3_) and trichloromethyl peroxy radical (·OOCCl_3_). These free radicals can bind with polyunsaturated fatty acids to produce alkoxy (R·) and peroxy radicals (ROO·) that, in turn, generate lipid peroxides that are highly reactive, change enzyme activity, and finally induce injury or necrosis with corresponding health problems [5,6].

CCl_4_ is known to decrease glutathione (GSH) of phase II enzymes and reduce antioxidant enzymes and substrates to induce oxidative stress, which is an important factor in acute and chronic injuries in various tissues [7,8]. Free radicals of CCl_4_ reduce the GSH contents and activities of antioxidant enzymes, leading to hepatic injury [9]. The depletions of these antioxidant enzymes occur secondary to the controlling action against peroxy radicals produced by CCl_4_. Reactive oxygen species cause oxidative DNA damage in the form of DNA adducts, genetic mutation, strand breakage, and chromosomal alterations [10]. Some recent investigations revealed that free radicals increased the number of argyrophilic nucleolar organizer regions (AgNORs) and activity of telomerase in kidney tissues [11], caused depletion of cytochrome P450 2E1, and increased the 8-hydroxyl-2 deoxyguanosine concentration [12]. DNA fragmentation causes p53 gene expression, blocks the cell cycle, and gives additional time to repair DNA. However, severe DNA damage triggers apoptosis [13].

*Sonchus asper* (SAME), locally called Mahtari, is a common herb that grows wildly and abundantly in open fields. Chemical studies of SAME indicated a high content of vitamin C (ascorbic acid) [14], carotenoids, and type ω-3 fatty acids [15,16]. Phenolic compounds, which are secondary metabolites in plants, are one of the most widely occurring groups of phytochemicals that exhibit a wide range of physiological properties, such as antioxidant, anti-allergenic, anti-microbial, anti-artherogenic, anti-thrombotic, anti-inflammatory, vasodilatory, and cardioprotective effects [17,18]. Chemical characterization of SAME has shown the presence of ionone derivatives of glycosides and sesquiterpene lactone glycosides [19]. These bioactive compounds have been shown to possess strong antioxidant and anti-inflammatory properties [20]. SAME has diuretic, refrigerant, sedative, and antiseptic properties that are used in the treatment of cough, bronchitis, and asthma [21] as well as kidney inflammation [22], and its decoction is used in the treatment of impotence (erectile dysfunction) [23]. With increasing recognition of herbal medicine and phytotherapy as alternative forms of health care, the objectives of this study were to evaluate the antioxidant and hepatoprotective properties of SAME against CCl_4_-induced hepatic injuries in Sprague–Dawley male rats.

## Methods

### Chemicals

Reduced glutathione (GSH), oxidized glutathione (GSSG), glutathione reductase, gamma-glutamyl p-nitroanilide, glycylglycine, bovine serum albumin (BSA), 1,2-dithio-bis nitro benzoic acid (DTNB), 1-chloro-2,4-dinitrobenzene (CDNB), reduced nicotinamide adenine dinucleotide phosphate (NADPH), CCl_4_, flavine adenine dinucleotide (FAD), glucose-6-phosphate, Tween-20, 2,6-dichlorophenolindophenol, thiobarbituric acid (TBA), picric acid, sodium tungstate, sodium hydroxide, trichloroacetic acid (TCA) and perchloric acid (PCA) were purchased from Sigma Chemicals Co. USA.

### Plant collection

Plants of *Sonchus asper* at maturity were collected from Wah Cantt, District Rawalpindi (Pakistan) during June, 2010. Plants were identified by Prof. Dr. Mir Ajab Khan (Dean Faculty of Biological Sciences, Quaid-i-Azam University Islamabad, Pakistan) and a specimen was submitted vide #147 at Herbarium of Pakistan (Quaid-i-Azam University Islamabad, Pakistan). Whole plant (leaves, stem, flowers, roots and seeds) were shades dried at room temperature for two weeks, chopped, ground mechanically of 1 mm mesh size.

### Preparation of plant extracts

Five kg powder of *Sonchus asper* was socked in 10 liters of methanol with shaking a number of times. After a week of socking the extract was filtered through Whatmann filter paper # 45, and the filtrate was evaporated through rotary vacuum evaporator to get 407 g of methanol crude extract (SAME), stored at 4°C until further use.

### Animals

Six week old, 36 Sprague Dawley male rats (190–200 g) were provided by National Institute of Health Islamabad and were kept in ordinary cages at room temperature of 25 ± 3°C with a 12 h dark/light cycle. They were allowed to standard laboratory feed and water. The study protocol was approved by Ethical Committee of Quaid-i-Azam University Islamabad for laboratory animal feed and care.

### Experimental design

To study the antioxidant effects of SAME, rats were equally divided into 6 groups (6 rats). Group 1 received only vehicles olive oil; (1 ml/kg bw) and DMSO (1 ml/kg bw) and have free access to food materials. Animals of group II, III and IV received CCl_4_ 3 ml/kg (30% in olive oil; v/v) intraperitoneally twice a week for four weeks. Group II was treated with CCl_4_ only while group III (100 mg/kg bw) and IV received (200 mg/kg bw) SAME (intragastric, in DMSO) after 48 h of CCl_4_. Rats of group V were given silymarin (intragastric, in DMSO) at a dose of 100 mg/kg bw, after 48 h of CCl_4_ treatment. Animals of a group VI were given SAME (200 mg/kg bw) intragastrically. Experimental period was of four weeks. After 24 h of the last treatment, all the animals were weighted, sacrificed; collected their blood, weighted and perfuse a liver in ice-cold saline solution. Half of liver tissues was treated with liquid nitrogen for further enzymatic and DNA damage analysis while the other portion was processed for histology.

### Assessment of serum markers

Serum analysis of various liver marker enzymes such as alanine aminotransferase (ALT), aspartate aminotransferase (AST), alkaline phosphatase (ALP), lactate dehydrogenase (LDH) and biochemical markers; level of total cholesterol (TC), high-density lipoproteins (HDL), low-density lipoproteins (LDL) and triglycerides (TG) were estimated by using standard AMP diagnostic kits (Stattogger Strasse 31b 8045 Graz, Austria).

### Assessment of antioxidant status

70 mg of liver tissue was homogenized in 10 volumes of 100 mmol KH_2_PO_4_ buffer containing 1 mmol EDTA (pH 7.4) and centrifuged at 12,000 × g for 30 min at 4°C. The supernatant was collected and used for determination of antioxidant status as described below using concentration of protein estimated following the method of Lowry et al. [24]. Antioxidant status, including activity of catalase [25], superoxide dismutase [26], glutathione-S-transferase assay [27], glutathione reductase [28], glutathione peroxidase [29], reduced glutathione assay [30] and lipid peroxidation assay [31] were performed on various hepatic samples.

### DNA fragmentation% assay

DNA fragmentation assay was conducted using the procedure of Wu et al. [32] with some modifications. The liver tissue (50 mg) was homogenized in 10 volumes of a TE solution pH 8.0 (5 mmol Tris–HCl, 20 mmol EDTA) and 0.2% triton X-100. 1.0 ml aliquot of each sample was centrifuged at 27,000 × g for 20 min to separate the intact chromatin (pellet, B) from the fragmented DNA (supernatant, T). The pellet and supernatant fractions were assayed for DNA content using a freshly prepared DPA (Diphenylamine) solution for reaction. Optical density was read at 620 nm with (SmartSpecTM Plus Spectrophotometer catalog # 170–2525) spectrophotometer. The results were expressed as an amount of % fragmented DNA by the following formula; % Fragmented DNA=T×100/(T+B)

### AgNORs count

Silver staining technique was used according to the Trere et al. [33]. The AgNORs technique was performed on dried slides as follows; unstained fixed slides were dewaxed by dipping for 3 minutes in xylene. After complete removal of wax, the slides were hydrated in decreasing ethanol concentration (90, 70 and 50%) and washed in distilled water for 10 min and dried in an oven. After drying slides were treated with one drop of colloidal solution (2% gelatin and 1% formic acid) and two drops of 50% AgNO_3_ solution on the slide and incubated at 35°C for about 8–12 min. The progressive staining was followed under a microscope to get golden colored nuclei and brown/black NORs. Then, the slide was washed in distilled water, treated for 1 min with 1% sodium thiosulphate at room temperature to stop the reaction, and washed in tap water. The cells were examined under a light microscope at 100 × magnifications and number of AgNORs was counted per cell.

### Histopathological studies

For microscopic evaluation, liver were fixed in a fixative (absolute ethanol 60%, formaldehyde 30%, and glacial acetic acid 10%) and embedded in paraffin, sectioned at 4 μm and subsequently stained with hematoxylin/eosin. Sections were studied under a light microscope (DIALUX 20 EB) at 40 and 100 magnifications. Slides of all the treated groups were studied and photographed.

### Statistical analysis

Data were expressed as mean and standard error (SE) and ANOVA test were used to analyze the difference among various treatments, with least significance difference (LSD) at 0.05 and 0.01 as a level of significance. SPSS ver. 14.0 (Chicago, IL, USA) and Microsoft Excel 2007 (Roselle, IL, USA) were used for the statistical and graphical evaluations.

## Results

### Effect of SAME on alanine aminotransferase, aspartate aminotransferase, alkaline phosphatase, and lactate dehydrogenase

The levels of certain serum marker enzymes, including alanine aminotransferase (ALT), aspartate aminotransferase (AST), alkaline phosphatase (ALP), and lactate dehydrogenase (LDH), are highly susceptible to hepatotoxins. They serve as markers of liver damage and oxidative stress, which promote the release of aminotransferases from hepatocytes into the bloodstream. The protective effects of SAME and silymarin on the activity of liver serum marker enzymes are shown in Table 1. Administration of CCl_4_ to rats significantly (P < 0.01) increased the activity of liver serum marker enzymes compared with controls. The levels of ALT, AST, ALP, and LDH were significantly (P < 0.01) and dose-dependently restored by treatment with SAME and silymarin compared with the CCl_4_ group. However, non-significant variation was observed by SAME treatment alone compared with the control group.

**Table 1:** Effect of SAME on liver function test in rat

**Treatment**	**ALT**	**AST**	**ALP**	**LDH (nmol/min/**
	**(U/L)**	**(U/L)**	**(U/L)**	**mg protein)**
Control	52. ± 2.12++	76 ± 2.74++	198 ± 3.93++	48.3 ± 2.38++
3 ml/kg CCl_4_	111 ± 3.42**	234.0 ± 4.27**	470.3 ± 6.49**	77.6 ± 2.46**
100 mg/kg SAME + CCl_4_	91 ± 3.23**++	136 ± 4.91**++	347.7 ± 10.7**++	66 ± 3.76**+
200 mg/kg SAME + CCl_4_	56 ± 1.35++	97.5 ± 2.90**++	217.8 ± 3.91*++	53 ± 2.61++
100 mg/kg Silymarin + CCl_4_	58 ± 2.68++	102.3 ± 4.99**++	227.5 ± 3.89**++	50 ± 4.08++
200 mg/kg SAME alone	50 ± 2.14++	75.5 ± 3.26++	195.3 ± 4.11++	46 ± 2.38++

Mean ± SE (n = 6 number).

** Significance from the control group at *P <* 0.01 probability level.

++ Significance from the CCl_4_ group at *P <* 0.01 probability level.

### Effect of SAME on triglycerides, total cholesterol, high-density lipoprotein, and low-density lipoprotein

The effect of SAME on the cholesterol profile is shown in Table 2. Administration of CCl_4_ significantly (P < 0.01) increased the concentration of triglycerides, total cholesterol, and low-density lipoprotein, but decreased the high-density lipoprotein level compared with the control group. The CCl_4_-induced alteration of the cholesterol profile was significantly (P < 0.01) restored with SAME treatment at both doses (100 mg/kg and 200 mg/kg) compared with the control group. Silymarin resulted in a degree of recovery of the cholesterol profile comparative with that achieved with the high dose of SAME.

**Table 2:** Effect SAME on serum level of biochemical markers in rat

**Treatment**	**Triglycerides (mg/dl)**	**Total cholesterol (mg/dl)**	**HDL (mg/dl)**	**LDL (mg/dl)**
Control	7.8 ± 0.45++	6.1 ± 0.25++	3.6 ± 0.21++	2.48 ± 0.32++
3 ml/kg CCl_4_	11.3 ± 0.58**	11.2 ± 0.23**	2.8 ± 0.18**	8.4 ± 0.17**
100 mg/kg SAME + CCl_4_	9.2 ± 0.41**++	7.7 ± 0.21**++	3.08 ± 0.09**++	4.2 ± 0.21**++
200 mg/kg SAME + CCl_4_	8.3 ± 0.18++	6.4 ± 0.27++	3.5 ± 0.20++	2..53 ± 0.35++
100 mg/kg Silymarin + CCl_4_	7.2 ± 0.44++	5.7 ± 0.19++	3.7 ± 0.21++	2.21 ± 0.31++
200 mg/kg SAME alone	7.5 ± 0.44++	5.8 ± 0.44++	3.75 ± 0.21++	2.36 ± 0.12++

Mean ± SE (n = 6 number).

** Significance from the control group at *P <* 0.01 probability level.

++ Significance from the CCl_4_ group at *P <* 0.01 probability level.

### Effect of SAME on antioxidant and lipid peroxidation enzymes in liver

The toxicity of CCl_4_ significantly decreased the liver catalase, superoxide dismutase, GSH S-transferase, GSH peroxidase (GSH-Px), and GSH reductase levels of the control group (Table 3). Addition of SAME at doses of 100 and 200 mg/kg and silymarin at 100 mg/kg reversed the decreased hepatic catalase and superoxide dismutase levels. In particular, the high dose of SAME (200 mg/kg) almost completely restored the catalase and superoxide dismutase activities to the control levels. The hepatic levels of GSH S-transferase, GSH-Px, and GSH reductase were partially reversed by SAME at 200 mg/kg.

**Table 3:** Effect of SAME on antioxidant defense system in rat liver

**Treatment**	**CAT**	**SOD**	**GST**	**GR **	**GSH-Px**
	**(U/min)**	**(U/mg protein)**	**nmol/min/mg protein**	**nmol/min/mg protein**	**nmol/min/mg protein**
Control	4.8 ± 0.10++	22.7 ± 1.18++	192 ± 2.49++	127.3 ± 10.5++	84.2 ± 5.9++
3 ml/kg CCl_4_	2.9 ± 0.06**	11.9 ± 0.38**	104 ± 2.4**	62.33 ± 9.28**	46 ± 3.6**
100 mg/kg SAME + CCl_4_	4.04 ± 0.09++	16.2 ± 0.38*++	142 ± 2.6*++	90.2 ± 11.3*++	61 ± 6.6*++
200 mg/kg SAME + CCl_4_	4.8 ± 0.125++	20.4 ± 0.37++	190 ± 2.9++	112.3 ± 6.5++	75 ± 4.2++
100 mg/kg Silymarin + CCl_4_	4.9 ± 0.116++	22.9 ± 0.57++	201 ± 2.5++	134.3 ± 5.7++	84 ± 4.0++
200 mg/kg SAME alone	4.96 ± 0.05++	24.7 ± 0.38++	199 ± 2.19++	132.3 ± 6.96++	87 ± 3.5++

Mean ± SE (n = 6 number).

*, ** Significance from the control group at *P <* 0.05 and *P <* 0.01 probability level.

++ Significance from the CCl_4_ group at *P <* 0.01 probability level.

### Effect of SAME on GSH and thiobarbituric acid-reactive substances in liver

The hepatic level of GSH was decreased while the thiobarbituric acid-reactive substances (TBARS) were increased in the CCl_4_-treated animals compared with the controls (Table 4). The CCl_4_-induced changes in these parameters were dose-dependently diminished by oral addition of various doses of SAME and silymarin. No significant changes in GSH or TBARS were found with SAME alone compared with control rats.

**Table 4:** Effect SAME on TBARS and GSH contents in rat liver

**Treatment**	**GSH (μmol/g tissue)**	**TBARS (nmol /min /mg protein)**
Control	1.23 ± 0.02++	28.33 ± 1.12++
3 ml/kg CCl_4_	0.60 ± .012**	52.17 ± 2.61**
100 mg/kg SAME + CCl_4_	1.09 ± 0.02**++	43.0 ± 2.76**++
200 mg/kg SAME + CCl_4_	1.81 ± 0.50++	30.0 ± 1.77++
100 mg/kg Silymarin + CCl_4_	1.27 ± 0.013++	27.17 ± 1.22++
200 mg/kg SAME alone	1.25 ± 0.018++	27.50 ± 1.82++

Mean ± SE (n = 6 number).

** Significance from the control group at *P <* 0.01 probability level.

++ Significance from the CCl_4_ group at *P <* 0.01 probability level.

### Effect of SAME on body weight, absolute liver weight, relative liver weight, % DNA fragmentation, and AgNORs in rat liver

The NORs stained by silver ion and the AgNOR-associated proteins, called AgNOR proteins, play a significant role in differentiation of tumor cells from normal cells, as shown along with increases in liver weight, relative liver weight, % DNA fragmentation, and % increases in body weight (Table 5). Administration of CCl_4_ significantly (P < 0.01) amplified the number of NORs per cell, increased the liver weight, increased the relative liver weight, increased the % DNA fragmentation, and decreased the % increase in body weight compared with untreated rats. However, 50 mg/kg of silymarin and various doses of SAME augmented the CCl_4_-induced intoxication and significantly (P < 0.01) recovered the NORs per cell, body weight, liver weight, and relative liver weight. The % DNA fragmentation was markedly (P < 0.01) decreased.

**Table 5:** Effect of SAME on absolute liver weight, relative liver weight, and % DNA fragmentation and AgNORs in rat liver

**Treatment**	**% increase in** **body weight**	**Absolute liver weight (g)**	**Relative liver weight (% to body weight)**	**AgNORs (NORs/cell)**	**%DNA fragmentation**
Control	35.00 ± 0.87++	7.0 ± 0.23++	0.07 ± 0.002++	2.0 ± 0.33++	5.33 ± 2.46++
3 ml/kg CCl_4_	24.52 ± 0.92**	8.6 ± .89**	0.86 ± .0016**	6.4 ± .29**	22.50 ± 3.68**
100 mg/kg SAME + CCl_4_	30.08 ± 0.81**++	7.4 ± 0.10*++	0.074 ± 0.001*++	3.5 ± 0.18**++	6.67 ± 2.08++
200 mg/kg SAME + CCl_4_	35.64 ± 0.73++	7.04 ± 0.23++	0.070 ± 0.0023++	2.14 ± 0.23++	5.67 ± 3.12++
100 mg/kg Silymarin + CCl_4_	36.82 ± 0.69++	6.83 ± 0.07++	0.068 ± 0.007++	1.9 ± 0.17**++	4.67 ± 2.23++
200 mg/kg SAME alone	35.72 ± 0.70++	6.8 ± 0.12++	0.068 ± 0.011++	1.96 ± 0.12++	5.50 ± 3.2++

Mean ± SE (n = 6 number).

*, ** Significance from the control group at *P <* 0.05 and *P <* 0.01 probability level.

++ Significance from the CCl_4_ group at *P <* 0.01 probability level.

### Effect of SAME on liver histology

**Figure 1:**
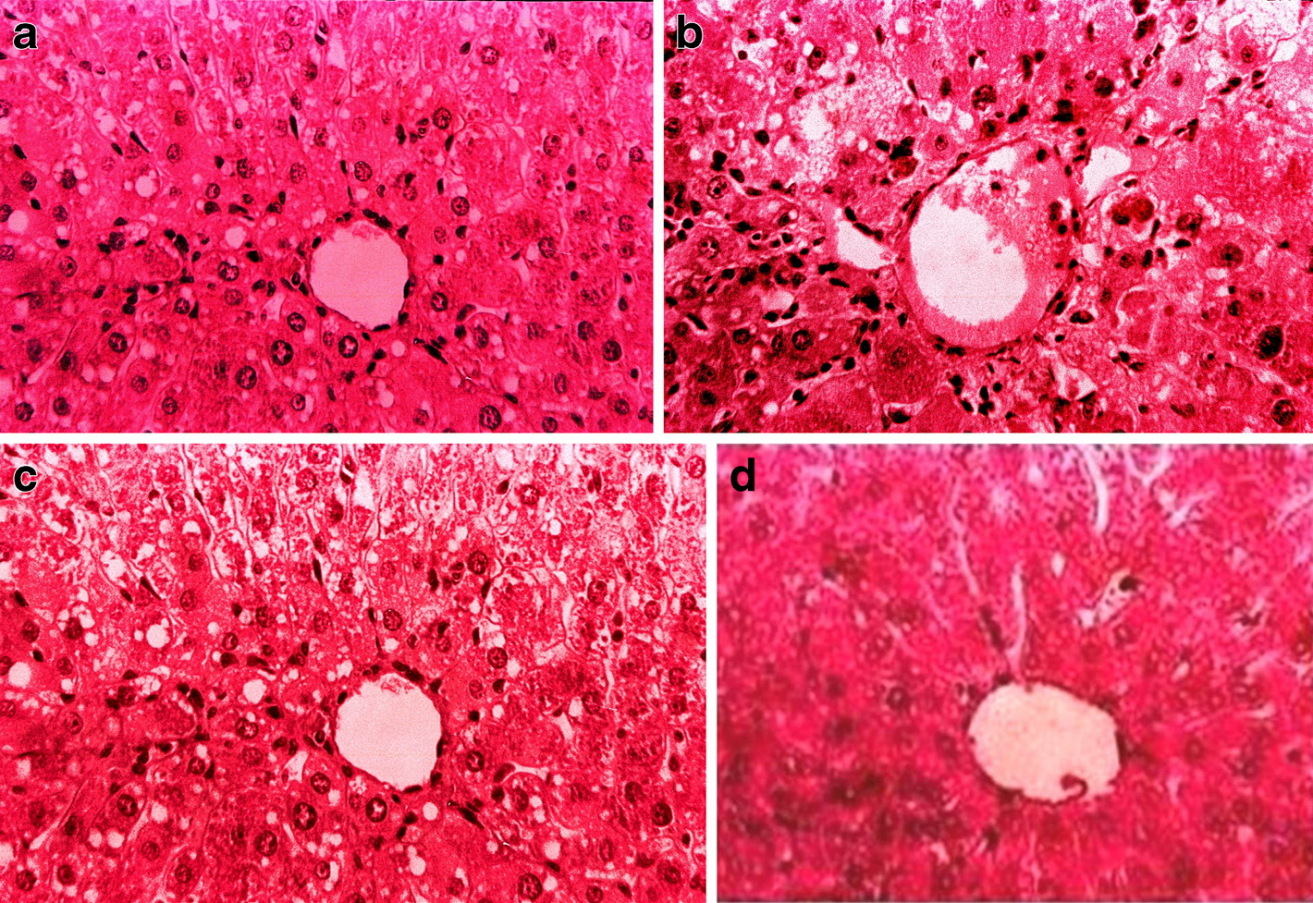
**(a) Hemotoxylin and Eosin-stained liver section of control rat (b) Liver section of CCl**_**4**_**treated rats showing marked fatty changes, cellular hypertrophy, degeneration of the lobular architecture and blood vessel congestion with disturbed epithelium (c) Liver section of CCl**_**4**_ **+ SAME showing less injuries (d) Liver section of CCl**_**4**_ **+ Silymarin showing less injuries.**.

Histopathological investigation results showed significant correlations with the biochemical study results. The graded observations of hematoxylin and eosin-stained sections are shown in Table 6 and Figure 1. Administration of CCl_4_ caused marked increases in fatty changes and cellular hypertrophy; cellular aggregation caused benign tumor formation, and inflammatory cell infiltration, ballooning, and congestion of blood vessels were observed compared with controls. Administration of SAME attenuated the hepatic injuries with little or no fatty changes; dilation of blood vessels and uniform hepatocyte morphology nearly identical to that of the control group was found. Similar observations were recorded after silymarin treatment. These results suggest a positive correlation of SAME and silymarin with the activities of serum aminotransferases and hepatic lipid peroxidation and hepatic antioxidant enzymes.

**Table 6:** Effects of SAME on histology of liver

**Treatment**	**Fatty changes**	**Cellular aggregation hypertrophy**	**Blood vessel congestion**	**Inflammatory cells infiltrations**
Control	-	-	-	-
3 ml/kg CCl_4_	+++	++	++	+++
100 mg/kg SAME + CCl_4_	-	−/+	-	−/+
200 mg/kg SAME + CCl_4_	-	-	-	-
100 mg/kg Silymarin + CCl_4_	-	-	-	-
200 mg/kg SAME alone	-	-	-	-

**-,** normal; +/−, mild; ++, medium; +++, severe disruption.

## Discussion

A number of drugs, toxic industrial chemicals, and viral infections have been reported to cause severe hepatic injuries, which are sometimes difficult to manage by medical therapies. It is important to evaluate plant extracts that can be used for improved treatment of hepatic failure caused by severe oxidative stress and necrosis [34]. Estimation of serum enzymes is a useful quantitative marker of the extent and type of hepatocellular damage. Increases in serum AST, ALT, ALP, and LDH levels have been attributed to damaged structural integrity of the liver, because these are cytoplasmic in location and are released into the circulation after autolytic breakdown or cellular necrosis [35]. Therefore, marked release of AST, ALT, ALP, and LDH into the circulation indicates severe damage to hepatic tissue membranes during CCl_4_ intoxication [36]. The reversal of increased serum enzymes in CCl_4_-induced liver damage by SAME may occur secondary to prevention of leakage of intracellular enzymes by the membrane stabilization and antioxidant activity of SAME; this was supported by the histological results in the present study. Thus, the anti-lipid peroxidation and/or adaptive nature of the systems brought about by SAME acted against the damaging effects of free radicals produced by CCl_4_. Similar hepatoprotective effects have been described for *S. arvensis*[37]. High levels of serum cholesterol, low-density lipoprotein, and triglycerides and lower concentrations of high-density lipoprotein were induced with CCl_4_ in rats, consistent with results of other studies [36,38,39].

Catalase, superoxide dismutase, and GSH-Px constitute a “mutually supportive team” of antioxidant defenses and play a key role in detoxification of free radicals [40,41]. Administration of CCl_4_ into rat livers increased lipid peroxidation, resulting in accumulation of superoxide radicals and consequently decreased their activities in the liver [42]. Our data revealed that CCl_4_ treatment significantly decreased the activities of catalase, superoxide dismutase, GSH-Px, and GSH reductase in liver tissues. Co-administration of SAME markedly decreased the toxicity of CCl_4_ and enzymatic activities. Ameliorating effects of different plant metabolites on these enzymes against the toxicity of CCl_4_ have also been documented [42]. TBARS is a major reactive aldehyde resulting from the peroxidation of polyunsaturated fatty acids. It is a useful indicator of tissue damage, including a series of chain reactions [43]. High hepatic levels of TBARS and nitrite with CCl_4_ treatment indicate extensive oxidative damage to liver tissues. Restoration of nitrite and TBARS with SAME may suggest a role of SAME in controlling various pathological conditions, including oxidative stress [20]. Histopathologic results in the present study revealed that CCl_4_ induces extensive fatty change, blood vessel congestion, cellular hypertrophy, necrotic foci, destruction of the lobular architecture, fibrosis, and nuclear degeneration in some areas, all of which were markedly diminished by induction of SAME*.* These data are in good agreement with the results of the serum aminotransferase activities and hepatic lipid peroxidation levels. Similar protection was evident with respect to necrosis, which is a more severe form of injury, in that it was minimized by treatment with SAME. Other studies on the protective effect of *Carissa opaca* extract against CCl_4_-induced hepatotoxicity in rats have revealed similar findings that are in agreement with the present findings [44].

## Conclusions

This study evaluated the pharmacological use of SAME against CCl_4_ induced liver injuries. Low lipid peroxidation and increased activities of various antioxidant enzymes indicate that SAME is able to protect various pathological conditions, including oxidative stress. It is suggested, to isolate the antioxidants from SAME that could be utilized as therapeutics against the oxidative diseases.

## Competing interests

The authors declare that they have no competing interests.

## Authors’ contributions

RAK made significant contribution to acquisition of data, analysis, drafting of the manuscript. MRK SS and NAS have made substantial contribution to conception and design, interpretation of data, drafting and revising the manuscript for intellectual content. All authors read and approved the final manuscript.

## Pre-publication history

The pre-publication history for this paper can be accessed here:

http://www.biomedcentral.com/1472-6882/12/90/prepub
